# Increased inflammation, endoplasmic reticulum stress and oxidative stress in endothelial and macrophage cells exacerbate atherosclerosis in ApoCIII transgenic mice

**DOI:** 10.1186/s12944-018-0867-5

**Published:** 2018-09-17

**Authors:** Han Yingchun, Ma Yahong, Wen Jiangping, He Xiaokui, Zhang Xiaohong

**Affiliations:** 1 Beijing Anzhen Hospital of Capital Medical University and Beijing Institute of Heart Lung and Blood Vessel Diseases, Beijing, 100029 China; 2 Department of Endocrinology, Beijing Puren Hospital, Beijing, 100062 China; 3 Department of Laboratory Medicine, Beijing Tongren Hospital, Capital Medical University, Beijing, 100730 China

**Keywords:** Apolipoprotein CIII, Triglyceride-rich lipoprotein, Endothelial cells, Macrophages, Inflammation, Endoplasmic reticulum stress, Oxidative stress

## Abstract

**Background:**

Overexpression of apolipoprotein CIII (ApoCIII) leads to hypertriglyceridemia (HTG) which promotes atherosclerosis development. However, it remains unclear whether ApoCIII affects the atherosclerosis alone by promoting the inflammation and endoplasmic reticulum (ER) stress, or in combination with HTG.

**Methods:**

Transgenic (ApoCIIItg) mouse models were used to investigate the atherogenic role of ApoCIII. Since endothelial cells and macrophages play crucial roles in atherosclerosis, we examined whether triglyceride-rich lipoproteins (TRLs), the major lipoproteins, in plasma of ApoCIIItg mice affect inflammation and ER stress levels in these cells. To further investigate the role of ApoCIII and triglyceride, we incubated HUVECs cells and peritoneal macrophages with TRLs with or without ApoCIII.

**Results:**

Increased inflammation and ER stress were found in the aorta of ApoCIIItg mice. TRLs increased ER stress and oxidative stress in HUVECs and macrophages in a dose dependent. Moreover, TRLs together with ApoCIII could induce a higher inflammation level than TRLs alone in these cells.

**Conclusions:**

Both TRLs and ApoCIII contribute to the progression of atherosclerosis, and the modulation of TRLs and ApoCIII may represent a novel therapeutic approach against HTG induced atherosclerosis.

## Background

Cardiovascular disease is a major cause of death globally. Atherosclerosis begins with vascular endothelial dysfunction, activation and recruitment of monocytes to the vascular wall, differentiation into macrophages, uptake of cholesterol and other lipoproteins, and formation of foam cells [1]. It is widely known that LDL, especially oxidative LDL, plays a major role in the initiation and development of atherosclerosis, and the molecular mechanism has also been studied extensively [2]. However, in recent years, it has become more evident that postprandial hyperlipidemia, large numbers of triglyceride-rich lipoproteins (TRLs), is also a significant risk factor for atherosclerotic cardiovascular disease [3–5]. Emerging evidence has shown that postprandial hyperlipidemia is a pro-inflammatory factor and TRLs participate in a large number of inflammation related processes, including excessive free radical production, leukocyte activation, endothelial dysfunction and the formation of foam cells [6, 7].

Regulation of plasma triglyceride homeostasis relies on a variety of enzymes and proteins including lipoprotein lipase (LPL), hepatic lipase, ApoCIII, ApoCII, and ApoAV [8]. An important regulator of triglyceride metabolism, the glycoprotein ApoCIII contains 79 amino acids, and is mainly synthesized by the liver and small intestine [9]. Plasma ApoCIII could inhibit LPL activity and interfere the liver uptake of TRLs by receptor dependent and independent pathways [10]. In addition, ApoCIII promotes the assembly and secretion of VLDL in the liver [11]. ApoCIII mainly distributes in TRLs at hypertriglyceridemia condition.

Surprisingly, in vitro experiments from our group [12] and others show that ApoCIII directly promotes the expression and activation of VCAM-1 in HUVECs and induces monocyte adhesion, thereby increasing the inflammatory response [10–12], indicating the significant effect of ApoCIII protein. Furthermore, two large genetic studies, one conducted in Denmark on people of mainly European origin [13] and the other in the United States on people of European and African origin [14], showed that people with loss-of-function mutations in the ApoCIII gene have a significantly reduced risk of having atherosclerotic cardiovascular disease.

Recently, we showed that ApoCIII induces hypertriglyceridemia which leads to aggravation of aortic atherosclerosis. Increased inflammation and oxidation level in vascular smooth muscle cells (VSMCs) play an important role in atherosclerosis progression [12]. Since both endothelial cells and macrophages are also involved in the initiation and development of atherosclerosis [15], we investigated the effect of TRLs to endothelial cells and macrophages and demonstrated the effect of triglycerides and ApoCIII in this process.

## Materials and methods

### Animals

The human ApoCIIItg, ApoCIII−/−, LDLR−/− and GPIHBP1−/− mouse models were purchased from the Jackson Laboratory (Bar Harbor, ME, USA). The study was approved by the Animal Care Committee of Peking University Health Science Center and in agreement with the Guide for the Care and Use of Laboratory Animals published by the US National Institutes of Health (NIH Publication, 8th Edition, 2011).

The ApoCIIItg/LDLR−/− and ApoCIIItg/GPIHBP1−/− mice were generated by crossing ApoCIIItg mice with LDLR−/− and GPIHBP1−/− Mice, respectively. The ApoCIII/GPIHBP1 DKO mice were generated by crossing ApoCIII−/− with GPIHBP1−/− mice.

Eight-week old male LDLR−/− (controls) and ApoCIIItg/LDLR−/− mice were fed with a high-fat, cholesterol-rich diet (20% lard and 0.5% cholesterol) for 3 months to develop atherosclerosis.

### Tissue harvesting and processing

After 3 months of pro-atherogenic diet, LDLR−/− (controls) and ApoCIIItgLDLR−/− mice were sacrificed. After the left ventricle after a right atrium cut was rinsed slowly with 1 × phosphate-buffered saline (PBS), the aorta was harvested for Western blotting. The heart was post-fixed with 4% paraformaldehyde for 2 h and immersed in 30% sucrose overnight. The top part of the heart was embedded in OCT, snap-frozen in liquid nitrogen, and stored at − 80 °C prior to sectioning. 10 μm cryosections of aortic sinus sections were then subjected to immunohistochemistry and ORO staining.

### Isolation of TRLs

Plasma of heparin-treated whole blood from ApoCIIItg/GPIHBP1−/− and ApoCIII/GPIHBP1 DKO mice was separated by centrifugation (4000 rpm, 4 °C, 10 min). TRLs, consisting of CMs and VLDLs, were isolated from plasma by ultracentrifugal spin (42,000 rpm, 10 °C, 3 h) at density 1.006 g/ml in a Hitachi P42AT rotor. Triglyceride contents in TRLs were measured by a kit from Sigma (TR0100).

### Cell culture

HUVECs cells were isolated from umbilical cords by collagenase digestion and cultured on plates coated with 50 mg/ml collagen as described [12]. Cells were maintained in M-199 medium supplemented with 20 mmol/L HEPES, pH 7.4, 20% fetal bovine serum (FBS), 5 ng/ml recombinant human fibroblast growth factor, antibiotics/antimycotics, and 90 mg/ml heparin (EC medium). Passages 3 to 5 were used and cell were treated when HUVECs were grown to 70–80% confluency. HUVECs were starved for 4 h before adding TRLs into the medium. For inflammation, ER stress or oxidative stress, HUVECs were treated with TRLs (100 μg/ml TG concentration or indicated concentration) for 24 or 48 h, and the cells were harvested. For inflammation, 1 μg/ml lipopolysaccharide (LPS) was added 6 h before RNA or protein extraction.

Female BALB/c mice (8–14 weeks of age) were intraperitoneally administered 2 ml of a 4.05% solution of Thioglycolate (Sigma, USA). Four days later, the mice were euthanized, and the peritoneal macrophages were collected in cold phosphate-buffered saline (PBS). The cells were cultured in Dulbecco’s modified Eagle’s medium (Gibco, USA) supplemented with 10% FBS and 2 mM l-glutamine.Two hours later, the medium was replaced with fresh medium to remove non-adherent cells. Fresh medium was added to the wells, and the cells were cultured at 37 °C for 24 h. To obtain sufficient numbers of cells, macrophages were collected from 2 to 3 mice and pooled for some experiments. Macrophages were treated with TRLs (100 μg/ml TG concentration) for 24 h.

Rat VSMCs were isolated from aortas of 80–100 g male Sprague-Dawley rats anesthetized intraperitoneally with sodium pentobarbital (50 mg/kg body weight). Rats were humanely sacrificed by cervical dislocation in order to obtain tissues to harvest VSMCs. All VSMCs experiments were performed on primary culture and passages 3–5 were used. VSMCs were treated with TRLs (100 μg/ml TG concentration) for 24 h.

### Immunohistochemistry (IHC)

For IHC analysis, cryosections were fixed (10 min) in cold 4% paraformaldehyde solution and rinsed (10 min) with PBS (0.1 M, pH 7.4) supplemented with 3% hydrogen peroxide. After washing and incubation (30 min) in blocking solution (PBS containing 10% goat serum), the sections were incubated overnight at 4 °C with 1:200 rabbit anti-VCAM-1 antibody (ab134047, Abcam), 1:400 rabbit anti-Mac2 antibody (sc-53,127, Santa Cruz) or 1:400 rabbit anti-4HNE antibody (ab46545, Abcam) diluted in blocking solution, washed three times with PBS, incubated with HRP-conjugated secondary antibody (for VCAM-1, Mac2, 4HNE) for 1 h at 37 °C, rinsed with PBS, counterstained with hematoxylin if needed and examined by light microscopy with diaminobenzidine (DAB) as chromogen.

### Western blot

Cells in culture plates were washed in ice-cold PBS twice and lysed in RIPA buffer (Cell Signaling Technology, #9806) containing complete protease inhibitor cocktail tablets (Roche). Protein lysates (20–40 μg) were resolved using SDS/PAGE and transferred to nitrocellulose membranes (Millipore). Primary antibody incubations were performed at 4 °C overnight using a 1:1000 dilution for anti-VCAM-1 antibody (ab134047, Abcam), anti-ICAM-1 antibody (ab171123, Abcam), or anti-GRP78 antibody (#3177, Cell Signaling Technology). Secondary antibody incubation was performed using a 1:5000 dilution of goat anti-rabbit HRP conjugate antibody (#7074, Cell Signaling Technology). Protein bands were visualized by LumiGLO® Reagent (#7003, Cell Signaling Technology).

### RNA isolation and quantitative real-time PCR

Total RNA from aorta and cells were extracted using Tri Reagent (Molecular Research Center), and first-strand cDNA was generated using an RT kit (Invitrogen). Quantitative real-time PCR was performed using primer sets shown in Table 1. Amplifications were performed in 35 cycles using an opticon continuous fluorescence detection system (MJ Research) with SYBR Green fluorescence (Molecular Probes, Eugene, OR). Each cycle consisted of heating denaturation for 30 s at 94 °C, annealing for 30 s at 55 °C, and extension for 30 s at 72 °C. All samples were quantified using the comparative C_T_ method for relative quantitation of gene expression, normalized to β-actin.

**Table 1:** Sequences of primers used for PCR

Gene	Primer sequences (5′ → 3′)
Human	
MCP1 (Forward)	GCTCATAGCAGCCACCTT
(Reverse)	GGAATCCTGAACCCACTT
Chop (Forward)	GGAAACAGAGTGGTCATTCCC
(Reverse)	CTGCTTGAGCCGTTCATTCTC
HO-1 (Forward)	TTTGAGGAGTTGCAGGAGC
(Reverse)	AGGACCCATCGGAGAAGC
GRP78 (Forward)	TCCTATGTCGCCTTCACT
(Reverse)	ACAGACGGGTCATTCCAC
GRP94 (Forward)	GTTTGGTGTCGGTTTCTA
(Reverse)	GAGTGTTTCCTCTTGGGT
β-actin (Forward)	CGTGGGCCGCCCTAGGCACCA
(Reverse)	TTGGCCTTAGGGTTCAGGGGGG
Mouse	
p47 (Forward)	ACACCTTCATTCGCCATATTGC
(Reverse)	TCGGTGAATTTTCTGTAGACCAC
p67 (Forward)	GCTGCGTGAACACTATCCTGG
(Reverse)	AGGTCGTACTTCTTCATTCTGTA
NOX-2 (Forward)	CCAAGGTATCCAAGTT
(Reverse)	TCCAGTCTCCCACAAT
NOX4 (Forward)	TAAGCCATCACCATCAT
(Reverse)	TGGAGGCAGTAGTAAATC
Catalase(Forward)	AGCGACCAGATGAAGCAG
(Reverse)	TTCCCACAAGATCCCAGT
SOD-1 (Forward)	TCCGTCGGCTTCTCGTCT
(Reverse)	ACCGCTTGCCTTCTGCTC
GRP78 (Forward)	ACTTGGGGACCACCTATTCCT
(Reverse)	GTTGCCCTGATCGTTGGCTA
IL-6 (Forward)	TTCTTGGGACTGATGCTG
(Reverse)	CTGGCTTTGTCTTTCTTGTT
TNF-α (Forward)	CTGTGAAGGGAATGGGTGTT
(Reverse)	CAGGGAAGAATCTGGAAAGGTC
IL-10 (Forward)	ACCTGGTAGAAGTGATGC
(Reverse)	AAGGAGTTGTTTCCGTTA
TGFβ-1 (Forward)	GGCGGTGCTCGCTTTGTA
(Reverse)	TCCCGAATGTCTGACGTATTGA

### Statistical analysis

All data are presented as mean ± SEM. Statistical comparison between two groups was performed using Student’s t-test or one-way ANOVA. A value of *P* < 0.05 was considered statistically significant.

## Results

### Inflammation, oxidative stress and ER stress in vivo

**Fig. 1:**
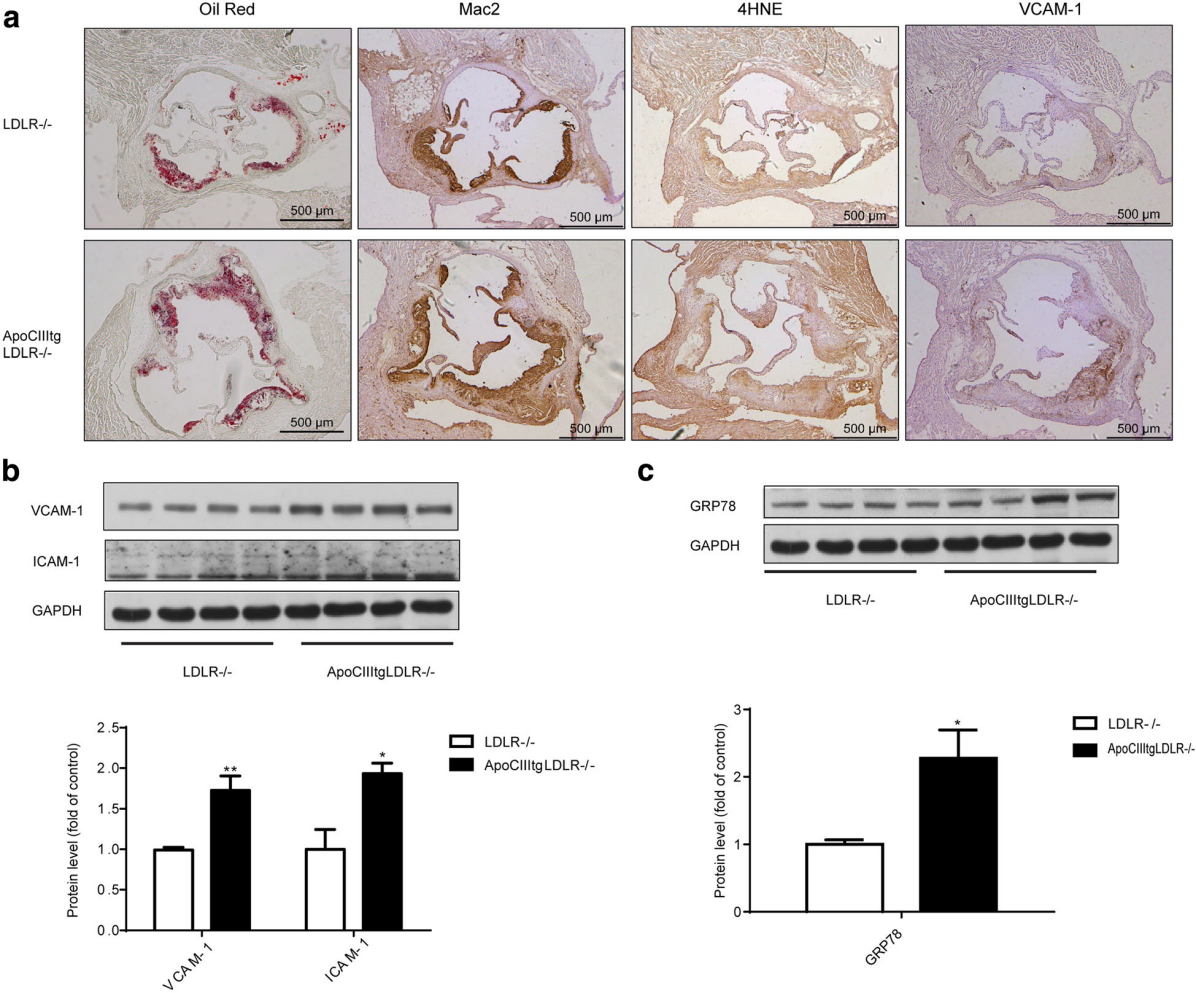
Increased inflammation, ER stress and oxidative stress in aorta of ApoCIIItgLDLR−/− mice comparing to LDLR−/− mice. (**a**) Representative images of Oil Red O (ORO) stained aortic roots and immunohistochemical staining of aortic sinus sections of Mac2, 4HNE and VCAM-1 expression in LDLR−/− and ApoCIIItgLDLR−/− mice. (**b**) Representative Western blot images of VCAM-1 and ICAM-1 protein expression in aortas of LDLR−/− and ApoCIIItgLDLR−/− mice and the protein quantification by densitometry (*n* = 4). (**c**) Representative Western blot images of GRP78 protein expression in aortas of LDLR−/− and ApoCIIItgLDLR−/− mice and the protein quantification by densitometry (*n* = 4). Values are expressed as mean ± SEM, *p < 0.05 **p < 0.01

To investigate the atherogenic role of ApoCIII, ApoCIIItg/LDLR−/− and LDLR−/− littermates were fed with an atherogenic diet for 12 weeks. Atherosclerotic lesion size in the aorta root was then measured by ORO staining. Consistent with our previous report [12], ApoCIII increases atherosclerotic lesions. Immunohistochemistry (IHC) staining for Mac2, a macrophage marker, indicated an increased macrophage infiltration in the lesion area. Staining of 4HNE, the product of lipid peroxidation that can be used to evaluate the oxidation level, showed that lipid peroxidation accumulated in the aorta of ApoCIIItg mice (Fig. 1a). IHC staining and Western blotting showed that VCAM-1 and ICAM-1 increased in aorta suggesting elevated inflammation (Fig. 1b). ER stress is involved in the initiation and development of atherosclerosis. We also detected that GRP78, an important chaperone molecule related to ER stress, significantly increases in the aorta (Fig. 1c). Thus, compared to the control LDLR−/− littermates, ApoCIIItg/LDLR−/− mice showed increased inflammation, oxidative stress and ER stress in local aorta.

### Inflammation in HUVECs and macrophages

**Fig. 2:**
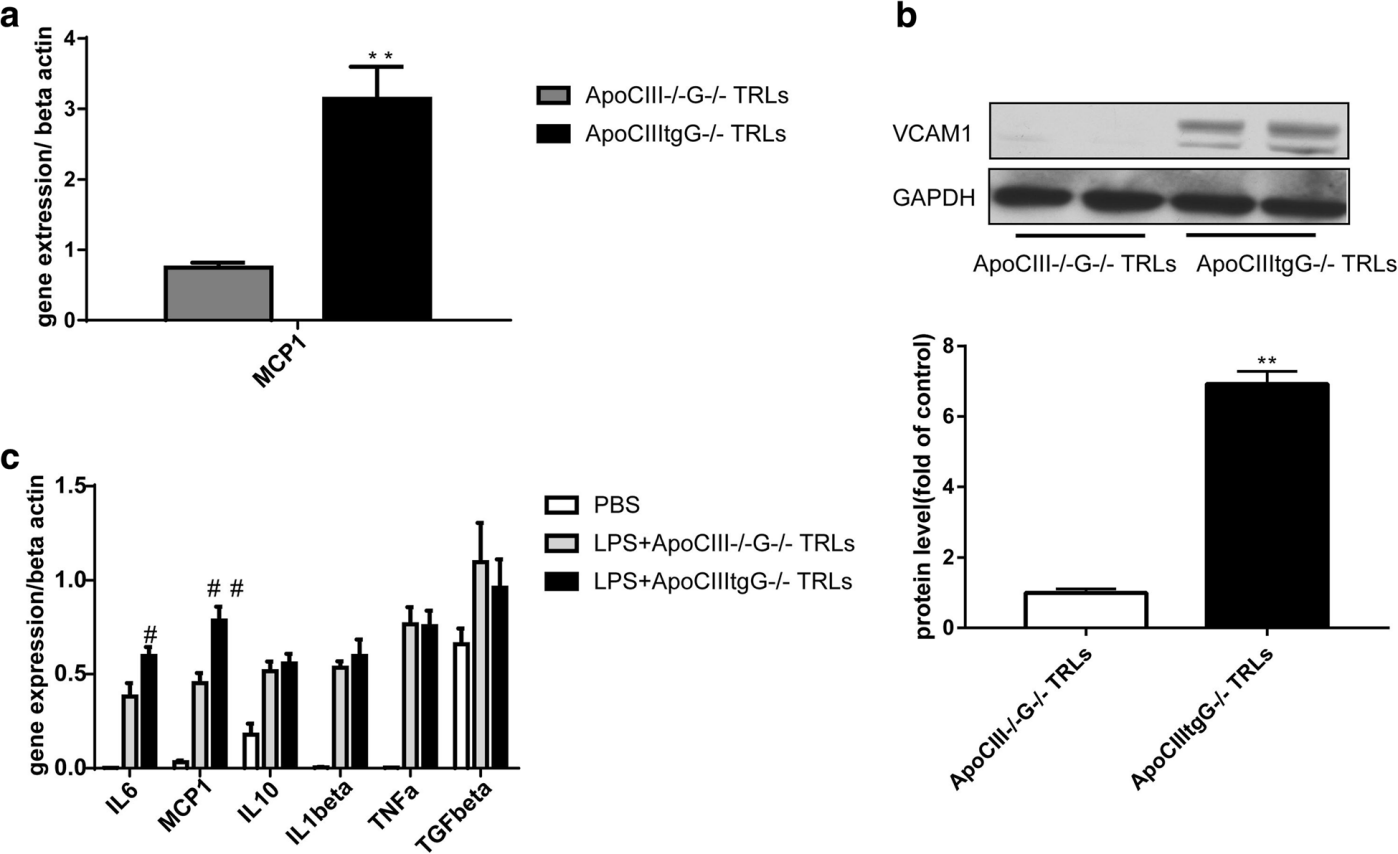
TRLs increase inflammation in HUVECs and macrophages dependent on ApoCIII. (**a**) MCP1 expression in HUVECs incubated with 100 μg/ml TRLs for 48 h (*n* = 4), **p < 0.01. (**b**) Western blot images (up) and the protein quantification (down) of VCAM-1 in HUVECs after the incubation with 20 μg/ml TRLs for 24 h and 1 μg/ml LPS for 6 h before the analysis (*n* = 4). (**c**) Expression of inflammation related genes in peritoneal macrophages after the incubation with 100 μg/ml TRLs for 24 h and an 1 μg/ml LPS stimulation for the last 6 h (*n* = 4)

Atherosclerosis is an inflammatory disease, starting from endothelial dysfunction, infiltration of macrophages in the vessel wall, and then the formation of foam cells [15]. ApoCIII transgene increased infiltration of macrophages and VCAM-1 expression, suggesting increased inflammation (Fig. 1a and b). It is reported that ApoCIII and VLDL containing ApoCIII could promote the expression of VCAM-1 and increase the adhesion of monocytes via a PKCbeta and NF-kB dependent pathway [6, 7]. Therefore, we examined whether authentic TRLs with or without ApoCIII have pro-inflammatory effects. Because the triglyceride level in the plasma of ApoCIII−/− mice is low, it is difficult to obtain the TRLs without ApoCIII (TRLs-ApoCIII). Therefore, we crossed ApoCIIItg mice and ApoCIII−/− mice with GPIHBP1−/− mice, another extreme hypertriglyceridemia mouse model, to generate two hyperlipidemia mice models: ApoCIIItgGPIHBP1−/− and ApoCIII/GPIHBP1 DKO mice, respectively. TRLs+/-ApoCIII were then separated from these two mice models. We found that TRLs+ApoCIII could induce MCP1 expression in HUVECs (Fig. 2a) and under the stimulation of 1 μg/ml LPS, TRLs+ApoCIII can also promote the expression of VCAM-1, reflecting the proinflammatory effects (Fig. 2b). Consistently, comparing to TRLs-ApoCIII, TRLs+ApoCIII promots the expression of IL6 and MCP1 also in peritoneal macrophages (Fig. 2c). These data suggested TRLs could aggravate inflammation depending on ApoCIII.

### ER stress and oxidative stress in HUVECs cells

**Fig. 3:**
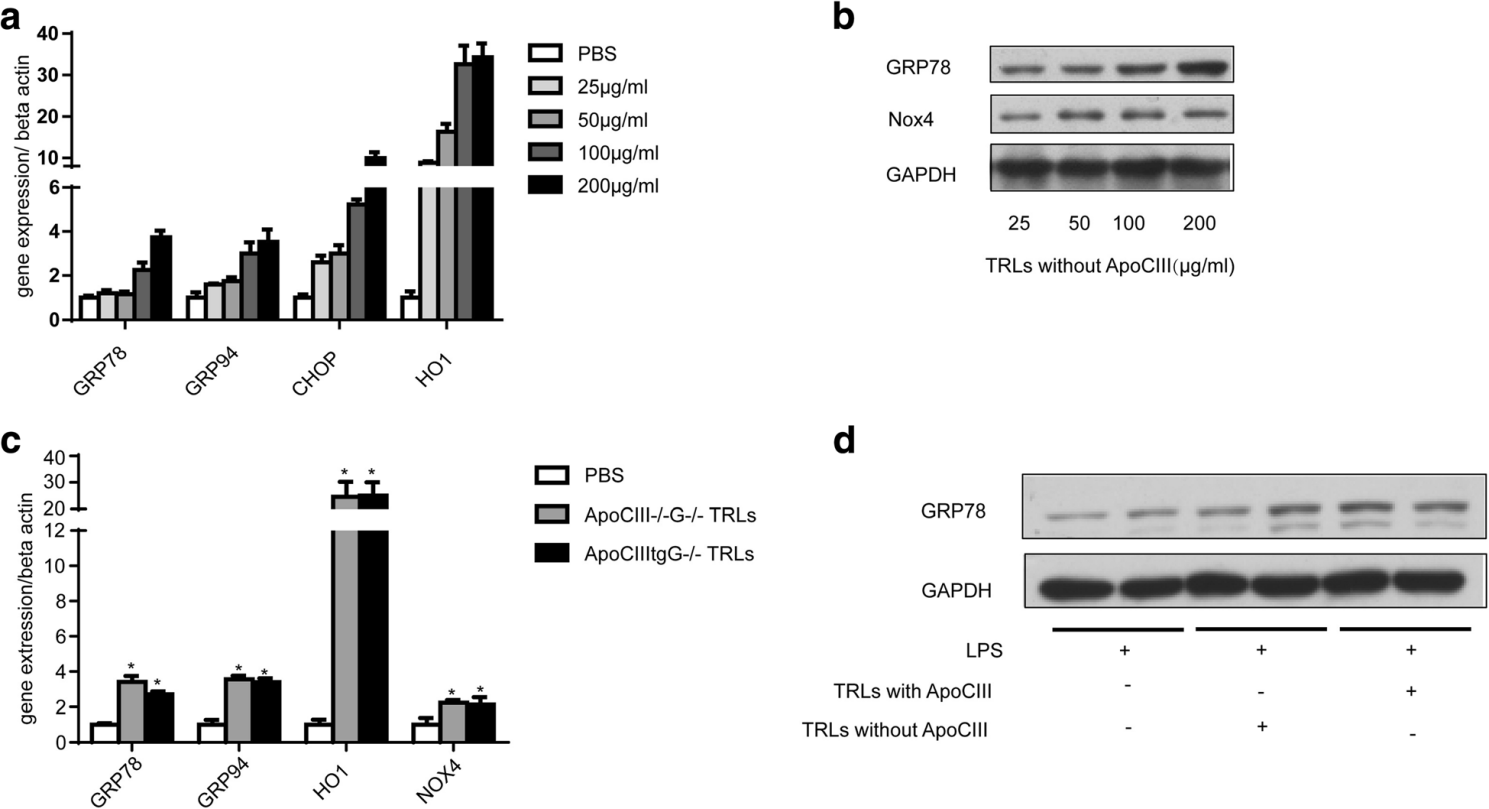
TRLs increase ER stress and oxidative stress in HUVECs independent of ApoCIII. (**a**) Expression of ER stress related genes in HUVECs after the incubation of different doses of TRLs without ApoCIII (*n* = 4). (**b**) Proteins related to ER stress in HUVECs after the incubation of different doses of TRLs without ApoCIII (*n* = 4). (**c**) Expression of ER stress related genes in HUVECs after the incubation of TRLs without or without ApoCIII (*n* = 4). *p < 0.05 vs. PBS. (**d**) Western blot images of GRP78 after the incubation of TRLs with or without ApoCIII (*n* = 4)

In vivo data showed increased expression of ER stress related proteins in ApoCIIItg mouse aorta (Fig. 1c). To investigate the effect of TRLs on ER stress in HUVECs cells, we used different doses of TRLs to incubate HUVECs. The result showed that TRLs-ApoCIII induces the expression of oxidative stress and ER stress related proteins in a dose-dependent fashion (Fig. 3a and b), although there was no significant difference between two types of TRLs (Fig. 3c and d). Therefore, our results established that TRLs increase oxidative stress and ER stress level independent of ApoCIII in HUVECs cells.

### ER stress and oxidative stress in macrophages

**Fig. 4:**
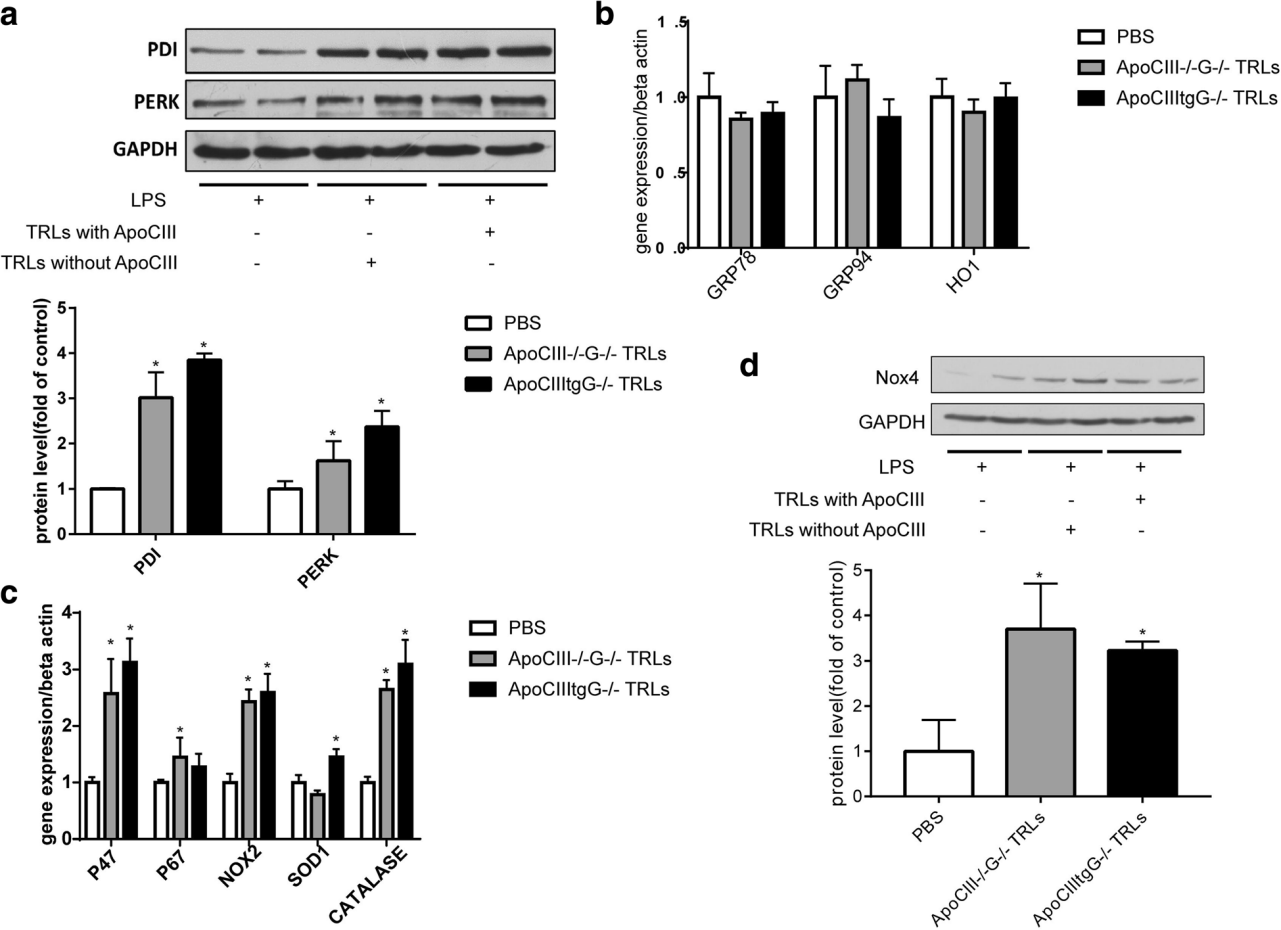
TRLs increase ER stress and oxidative stress in macrophages and VSMCs independent of ApoCIII. (**a**) Western blot images (up) and the protein quantification (down) of PDI and PERK in VSMCs after 48 h incubation with TRLs (100 μg/ml TG concentration). Expression of ER stress related genes (**b**) and oxidative stress related genes (**c**) in peritoneal macrophages after the incubation with TRLs (*n* = 4), *p < 0.05 vs. PBS. (**d**) Western blot images (up) and the protein quantification (down) of Nox4 in macrophages after the incubation of TRLs with or without ApoCIII (*n* = 4)

To investigate the effect of TRLs on oxidative stress and ER stress in macrophages, we incubated rat VSMCs and mouse peritoneal macrophage cells with TRLs. TRLs+/-ApoCIII markedly increased the protein expression of PDI (protein disulfide isomerase) and PERK in VSMCs, but there was no significant difference between the two types of TRLs (Fig. 4a). Similarly, both types of TRLs promote the expression of the proteins related to oxidative stress in macrophage cells, and there was no significant difference between these two TRLs types (Fig. 4b). However, unlike endothelial cells and VSMCs, no TRLs could induce the expression of GRP78, GRP94, and HO-1 genes, suggesting that TRLs have no effect on ER stress in macrophages (Fig. 4c and d).

## Discussion

Zilversmit first proposed that postprandial hyperlipidemia may play an important role in atherosclerosis thirty years ago [16]. In recent years, lines of evidence from epidemiological investigations, clinical and experimental research emerged showing that non-fasting TRLs level as a risk factor for atherosclerosis and cardiovascular disease [17]. “Residual risk” in statin therapy also supports TRLs as a risk factor in atherosclerosis [18].

TRLs can indirectly promote atherosclerosis. Hypertriglyceridemia is often associated with the atherosclerosis prone lipid profile. Increased small dense LDL is much more easily oxidized and adhered to the vessel wall, and is harder to remove; and increased lipid transferring to TRLs from HDL leads to lower HDL cholesterol [19, 20]. Elevated triglycerides, small dense LDL and reduced HDL form the lipid distribution of atherosclerosis.

TRLs can also directly promote atherosclerosis. It has shown that remnant like lipoprotein (RLP) in human atherosclerotic plaques and chylomicron remnants (CMR) enter and remain in the blood vessel wall. CMR and TRLs contribute to endothelial dysfunction, macrophage foam cell formation, and the proliferation of vascular smooth muscle cells [21]. TRLs can increase the expression of coagulation factor VII and plasminogen activator inhibitor I (PAI-1) therefore promoting coagulation, including platelet aggregation [22].

As the dominating apolipoprotein in TRLs, whether ApoCIII directly promotes atherosclerosis still remains to be determined. Here, to separate the effect of ApoCIII and TRLs, we isolated biologically active TRLs+/-ApoCIII from the plasma of ApoCIIItgGPIHPB1−/− and ApoCIII/GPIHBP1 DKO mice. Our previous data showed that TRLs could induce proliferation and inflammation in VSMCs dependent on ApoCIII [12]. In the present study, we mainly focused on the endothelial cells and macrophages.

In vivo immunohistochemistry and Western blot experiments showed that ApoCIII overexpression increases the inflammation level in aorta. In our in vitro experiments, we used the natural TRLs+/-ApoCIII and confirmed that ApoCIII can promote the expression of inflammatory factors in endothelial cells and macrophages. The pro-inflammation effect of ApoCIII involves the Akt pathway in VSMCs as we reported previously [12], and the mechanism of ApoCIII in endothelial cell needs to be investigated in the future.

Compared to control mice, ApoCIIItg mice showed higher ER stress level in aorta (Fig. 1c). Incubated with TRLs, HUVECs showed dose dependent increase of ER stress level (Fig. 3a and b). However, there was no difference between effects of TRLs with and without ApoCIII (Fig. 3c and d). It has been reported that TRLs induce ER stress and oxidative stress in endothelial cells [23]. Here, we confirmed that, unlike inflammation, ER stress and oxidative stress are induced by TRLs independent of ApoCIII.

A targeted approach to reduce plasma levels of ApoCIII can be achieved by providing an antisense inhibitor of ApoCIII synthase (ApoCIII-ASO) [24–26]. When people with severe hypertriglyceridemia were treated with ApoCIII-ASO, the resulting decrease of ApoCIII in the plasma was accompanied by a major reduction of triglyceride in the plasma and a substantial increase of HDL-C [25]. However, the effects of inhibiting ApoCIII synthesis on ASCVD risk are not known.

## Conclusions

In conclusion, our data showed that ApoCIII promotes inflammation in endothelial cells and macrophages and that TRLs from ApoCIII induced HTG could lead to high ER stress level in endothelial cells which may contribute to the progression of atherosclerosis. These results suggest that ApoCIII-ASO may represent a novel therapeutic approach against ASCVD.

## Abbreviations

*4HNE*: 4-Hydroxynonenal
*ApoAV*: Apolipoprotein AV
*ApoCII*: Apolipoprotein CII
*ApoCIII*: Apolipoprotein CIII
*ApoCIII-ASO*: ApoCIII-antisense oligonucleotide
*ASCVD*: Arteriosclerotic cardiovascular disease
*CHOP*: CCAAT/enhancer-binding protein homologous protein
*CMR*: Chylomicron remnants
*CMs*: Chylomicrons
*DAB*: Diaminobenzidine
*DKO*: Double knockout
*ER*: Endoplasmic reticulum
*FBS*: Fetal bovine serum
*GAPDH*: Glyceraldehyde 3-phosphate dehydrogenase
*GPIHBP1*: Glycosylphosphatidylinositol high density lipoprotein-binding protein1
*GRP78*: Glucose regulated protein 78
*GRP94*: Gloucose requlated protein 94
*HO-1*: Heme oxygenase-1
*HTG*: Hypertriglyceridemia
*HUVECs*: Human umbilical vein endothelial cells
*ICAM-1*: Intercellular Adhesion Molecule 1
*IL1*: Interleukin 1
*IL10*: Interleukin 10
*IL6*: Interleukin 6
*LDL*: Low density lipoprotein
*LDLR*: Low density lipoprotein receptor
*LPL*: Lipoprotein lipase
*LPS*: Lipopolysaccharide
*MCP1*: Monocyte chemotactic protein 1
*Nox4*: NADPH oxidase 4
*OCT*: Optimal cutting temperature
*ORO*: Oil red O
*P47*: NADPH oxidase p47
*P67*: NADPH oxidase p67
*PBS*: Phosphate-buffered saline
*PDI*: Protein disulfide isomerase
*PERK*: Protein kinase R (PKR)-like endoplasmic reticulum kinase
*PKC*: Protein kinase C
*RLP*: Remnant like lipoprotein
*SOD1*: Superoxide dismutase1
*TGFbeta*: Transforming growth factor-β
*TNF*: Tumor Necrosis Factor
*TRLs*: Triglyceride-rich lipoproteins
*VCAM-1*: Vascular cell adhesion molecule 1
*VLDL*: Very lowdensity lipoproteins
*VSMCs*: Vascular smooth muscle cells
